# Experiences of intensive care unit nurses working with COVID-19 patients: A systematic review and meta-synthesis of qualitative studies

**DOI:** 10.3389/fpubh.2022.1034624

**Published:** 2022-11-15

**Authors:** Alireza Nikbakht Nasrabadi, Soheila Abbasi, Abbas Mardani, Maryam Maleki, Zeljko Vlaisavljevic

**Affiliations:** ^1^School of Nursing and Midwifery, Tehran University of Medical Sciences, Tehran, Iran; ^2^Pediatric and Neonatal Intensive Care Nursing Education Department, School of Nursing and Midwifery, Tehran University of Medical Sciences, Tehran, Iran; ^3^Nursing and Midwifery Care Research Center, Department of Medical Surgical Nursing, School of Nursing and Midwifery, Iran University of Medical Sciences, Tehran, Iran; ^4^University Clinical Center of Serbia, Clinic for Gastroenterology and Hepatology, Belgrade, Serbia; ^5^Department of Nursing, Medical School of Vocational Studies Medika, Belgrade, Serbia

**Keywords:** nurse, intensive care unit, COVID-19, experience, meta-synthesis

## Abstract

**Objectives:**

Intensive Care Unit (ICU) nurses are at the forefront of fighting and treating the Coronavirus 2019 (COVID-19) pandemic and are often directly exposed to this virus and at risk of disease, due to their direct care for infected patients. This study aims to synthesize the experiences of ICU nurses working with COVID-19 patients.

**Methods:**

A systematic review and meta-synthesis of qualitative studies were undertaken. A systematic literature search in four databases, including Web of Sciences, Scopus, Embase, and PubMed (including Medline), was performed. Original qualitative studies and the qualitative section of mixed method studies, written in English, which focused on the experiences of only ICU nurses working with COVID-19 patients, were included.

**Results:**

Seventeen qualitative studies and two mixed-method studies were included in the review. As a result of the inductive content analysis, six main categories were identified, as follows: “distance from holistic nursing,” “psychosocial experiences,” “efforts for self-protection and wellbeing,” “organizational inefficiency,” “job burnout,” and “emerging new experiences in the workplace.”

**Conclusions:**

The findings from this study suggest that healthcare authorities and policymakers can facilitate the provision of high-quality patient care during the COVID-19 pandemic through appropriate planning to provide adequate support and training, prevent shortages of nursing staff and equipment, and provide adequate attention to the psychological needs and job satisfaction of ICU nurses.

**Systematic review registration:**

https://www.crd.york.ac.uk/prospero/display_record.php?RecordID=256070, identifier: CRD42021256070.

## Introduction

Viral infectious diseases have always been a threat to human health and survival. Middle East Respiratory Syndrome (MERS), Severe Acute Respiratory Syndrome (SARS), and the current Coronavirus 2019 (COVID-19) are the three global viral infectious diseases that have occurred worldwide in the last two decades (1). COVID-19 emerged in Wuhan, Hubei Province of China, in December 2019. In just a few months, the disease was declared a pandemic by the World Health Organization (WHO), in March 2020 (2). COVID-19 has many unknown clinical dimensions and is related to SARS-CoV-2 (3). It is proven that the disease is transmitted from person to person and causes symptoms from mild upper respiratory tract infection to severe respiratory failure and even death (4).

Pandemic diseases have a huge impact on healthcare systems, especially within the workforce (5). COVID-19 has created many challenges among healthcare workers (HCWs), due to its special characteristics, such as high prevalence, being unknown, and endangering the lives of HCWs (3). Healthcare professionals stand at the forefront of pandemic diseases (6). Nurses, as the largest workforce in the health care system, play an essential role in high-quality patient care (7). Nursing is one of 40 professions with a high prevalence of job stress, according to the National Association of Safety Professionals in the United States (7). Nurses, and especially Intensive Care Unit (ICU) nurses, are at the forefront of fighting and treating pandemic diseases, they are often directly exposed to these viruses, and are at risk of disease, due to the direct care they provide for infected patients (5, 8).

Patients infected with COVID-19 may need intensive care (9). Among the hospital wards, the ICU is one equipped with sophisticated equipment which is there to provide intensive care and comprehensive services to patients with life-threatening conditions (10). Nurses working in the ICU have a wide variety of duties and responsibilities, including constant attention to patients' needs, decision-making in critical situations, and interaction with patients' families. In addition, they spend more time in direct patient care than in other wards (7, 11). The results of the study by Abbey et al. showed that ICU nurses perform 3,081 different activities during the day, including direct care, indirect care, personal activities and unit-related activities, of which 43% are performed simultaneously (12). Therefore, ICU nurses experience a heavy workload (13).

The COVID-19 pandemic has been posing an unprecedented and difficult challenge for ICU nurses (14). Witnessing death and exposure to illness, stigma, job stress (such as lack of resources, redeployment, poor organizational support), isolation from loved ones due to concerns about transmission of the disease, lack of ICU beds, multiple end-of-life decisions, lack of adequate personal protective equipment (PPE), and loneliness are risk factors that can put nurses in a state of mental and physical stress which profoundly affects their wellbeing and mental health (14–17).

Dyspnea, chest discomfort, palpitations, headache, nausea, and dizziness are some of the common symptoms reported by ICU nurses in the COVID-19 pandemic (18). In addition, a nationwide survey of 726 ICU nurses in the COVID-19 pandemic found that ICU nurses experience symptoms of anxiety (27%), depression (18.6%), and post-traumatic stress disorder (22%) (14). The results of a qualitative study in China showed that nurses caring for COVID-19 patients experienced negative physical effects and emotions, such as fatigue, helplessness, discomfort from high-intensity work, anxiety, and worry for patients and their families (15).

Knowing the experience of nurses caring for patients with COVID-19 is critical to improving patient safety, quality of care, and the work environment of staff during future pandemics (18). In this regard, studies have examined the experiences of ICU nurses during the COVID-19 pandemic. Nevertheless, to our knowledge, there is no study that has synthesized these experiences. Therefore, the aim of this systematic review was to synthesize the experiences of ICU nurses working with COVID-19 patients.

## Methods

### Protocol and registration

The present systematic review is a meta-synthesis of qualitative studies. Meta-synthesis is a method that synthesizes qualitative studies with an interpretive approach (19, 20). The purpose of such methods is to obtain an increasing volume of qualitative research, gather a wide range of participants' experiences, and improve healthcare by facilitating knowledge transfer (20). Therefore, this has allowed the authors to integrate and synthesize the experiences of ICU nurses working with COVID-19 patients from qualitative studies in order to create comprehensive knowledge and understanding. The Preferred Reporting Items for Systematic review (PRISMA) flow chart was applied as a guideline for finding and selecting all qualitative studies (21). The protocol of this systematic review has been registered on the PROSPERO under the code - CRD42021256070: https://www.crd.york.ac.uk/prospero/display_record.php?RecordID=256070.

### Search process and eligibility criteria

Discussions were held between the members of the research team to identify the appropriate keywords. They also performed a pilot search of specialized and general databases to find relevant keywords. The Boolean search method was used to identify articles related to the experiences of ICU nurses working with COVID-19 patients, using the following keywords: [(Nurse^*^ OR “healthcare worker” OR “healthcare provider” OR “healthcare team” OR “healthcare personnel” OR caregivers OR “health worker” OR “healthcare profession”) AND (Experience OR “lived experience” OR “reported experience” OR “personal experience”) AND (COVID-19 OR “coronavirus disease 2019” OR “coronavirus pandemic” OR “SARS-CoV-2” OR “COVID-19 crisis” OR “COVID-19 outbreak”) AND (“Qualitative study” OR “Qualitative research” OR “exploratory research” OR “exploratory study”)]. Accordingly, the online databases of Web of Sciences, Scopus, Embase, and PubMed (including Medline) were searched up to August 2022, without time limiting for extracting articles published in online peer-reviewed scientific journals. The researchers searched the bibliographic cross-references to improve search coverage. Inclusion criteria for selecting relevant studies included qualitative studies, qualitative sections of mixed method studies, focusing on the experiences of only ICU nurses caring for patients with COVID-19, and published in peer-reviewed scientific journals. The exclusion criteria were the following: quantitative articles, articles without exact relevance to the experience of ICU nurses working with COVID-19 patients, studies focusing on the experiences of other healthcare professionals in the care of patients with COVID-19, and studies focusing on ICU nurses' experiences along with other professionals.

### Study selection

Databases were searched by using predetermined keywords. The authors (MM and SA) independently screened the titles and abstracts of the studies retrieved during the search process. The results were shared among researchers *via* EndNote software to make final and collective decisions about the inclusion and exclusion of studies. The authors conducted online conversations to share search results and decide on the next steps of the study, resolve disagreements, and reach a consensus on the inclusion of selected studies. Once all eligible studies had been selected, details from each paper in the pre-piloted data extraction table were extracted and recorded.

### Quality appraisal

The quality of qualitative articles and the qualitative section of two mixed-method studies was evaluated using the Critical Appraisal Skills Programme (CASP) tool (22). The CASP tool contains ten questions and is a common tool for examining the limitations and strengths of any qualitative research method (22). Studies based on this tool are classified into three levels in terms of quality: high, medium, and low. Studies with 8 to 10 criteria are in the high-quality category, studies with 5 to 7 criteria are in the medium-quality category and those with 4 criteria or less fall into the category of low quality (23). The two authors (MM and SA) independently evaluated the quality of the articles using the CASP tool; in case of any discrepancies, these were discussed and resolved by a third author (ANN). Any disagreements and discrepancies between the authors were resolved by consensus.

### Data collection process and synthesis of results

A data extraction table, including the first author's surname, year of publication, country of study, the purpose of study, design, sample size and settings, sampling method, method of data collection, and type of data analysis was developed.

Methods for analyzing data in meta-synthesis were varied, in order for the number of analysis methods to match the number of authors. We used the guidelines provided by Lachal et al. for data analysis (20). The first step in this process involved carefully reading and rereading each study in order to appraise, familiarize, identify, extract, record, organize, compare, and relate. In this step, the two authors independently read each study carefully and extracted citations from the findings/results section of each study. The second stage of the process was coding. In this step, the two researchers independently encoded each part of the data extracted in the first step (all studies) and performed a line-by-line coding. The third step involved grouping the codes and categorizing them into a hierarchical tree structure. In this step, the themes in the articles were compared to match the themes of one article with the themes of the other articles, while ensuring that the key theme took the same themes from different articles. In addition, the authors obtained a list of descriptive themes very close to the data. Finally, the last stage, which is considered the most subjective stage of analysis, was the generation of analytical themes. Using the inductive content analysis and following the initial immersion in the data by reading and re-reading, final themes and sub-themes were formed (20).

### Data trustworthiness

The authors used several strategies to ensure the trustworthiness of the data. Two reviewers reached a consensus at each step, before proceeding to the next step. In addition, the data analysis process was confirmed by two qualitative researchers, as peer checking. Moreover, triangulation enhances the trustworthiness of the data. Triangulation is somewhat different in the context of meta-synthesis. It involves the use of findings from qualitative research studies related to the research question. Accordingly, throughout the meta-synthesis process, a triangulation approach was maintained by comparing each included study to discover a new understanding of the experiences of ICU nurses working with COVID-19 patients. Furthermore, the researchers were experts or trained in meta-synthesis and conducting qualitative research and analysis (24–26).

## Results

### Search outcome and selection of studies

**Figure 1:**
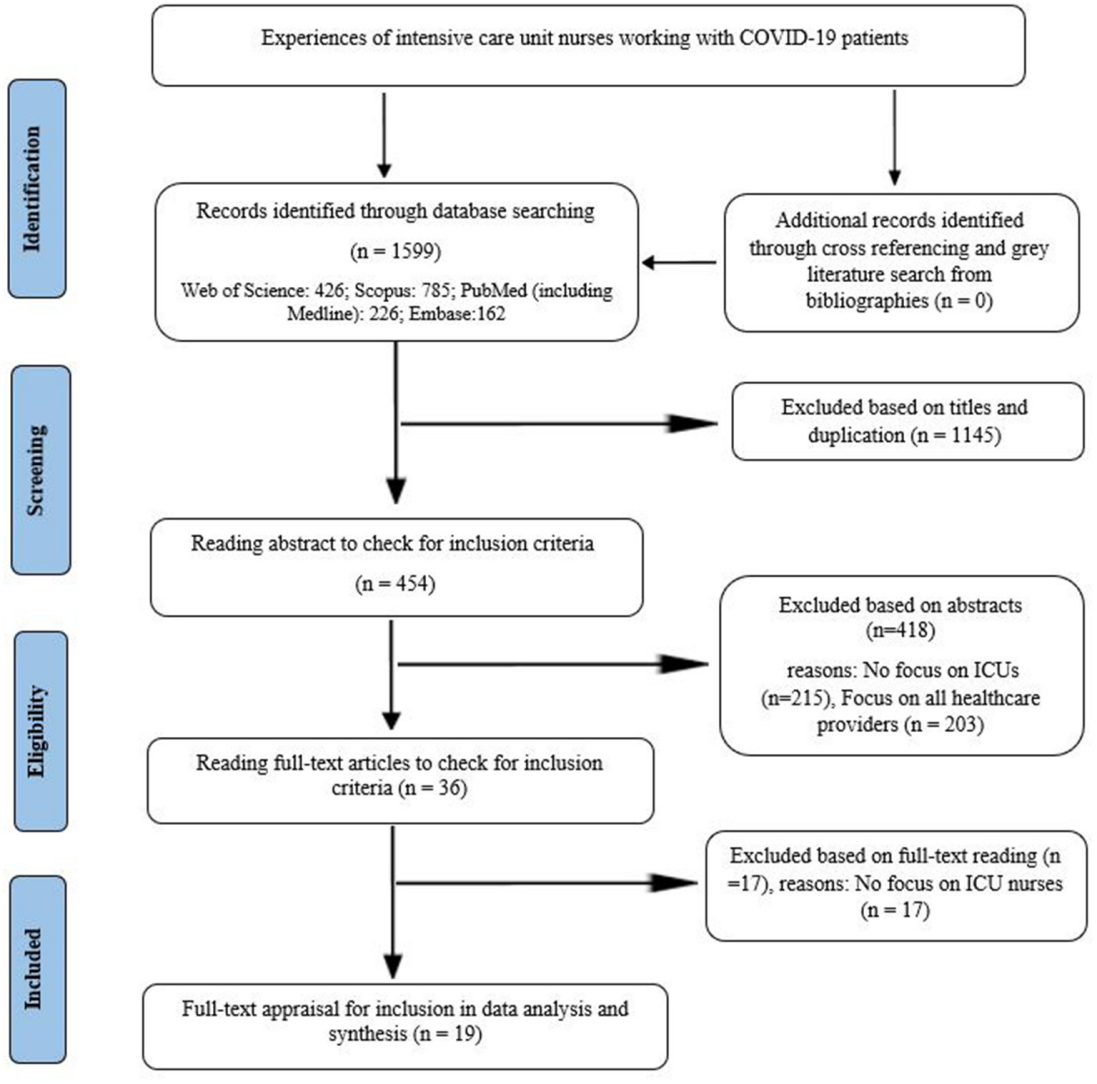
The preferred reporting items for systematic reviews and meta-analyses (PRISMA).

The results of our search in four databases are presented in Figure 1. In the search process, which was performed using predefined keywords, 1,599 articles were retrieved. After removing duplicate and unrelated titles, and reading the abstract and full text, nineteen studies were finally selected for data synthesis. No further studies were found for inclusion in the meta-synthesis during the review of the references list of selected studies. The authors evaluated the quality of the selected studies during the full-text appraisal phase (Table 1). No studies were excluded from the meta-synthesis process based on their quality. The study flow chart, according to the reported preferential cases for systematic reviews and meta-analysis (PRISMA), is presented in Figure 1.

**Table 1:** Critical appraisal skills program (CASP) result.

**References**	**A**	**B**	**C**	**D**	**E**	**F**	**G**	**H**	**I**	**J**	**Score**
Anderson et al. (27)	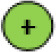	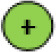	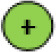	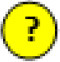	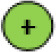	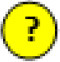	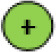	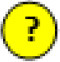	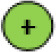	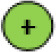	7/10
Aydin et al. (28)	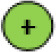	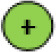	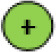	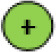	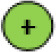	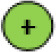	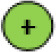	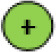	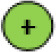	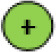	10/10
Bergman et al. (29)	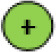	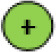	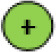	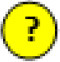	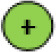	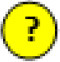	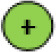	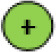	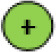	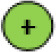	8/10
Cadge et al. (30)	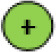	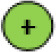	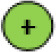	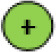	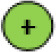	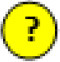	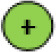	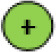	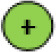	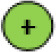	9/10
Chegini et al. (31)	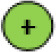	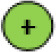	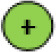	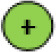	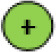	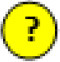	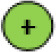	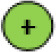	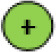	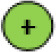	9/10
Conz et al. (32)	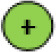	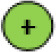	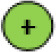	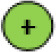	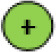	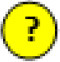	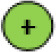	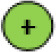	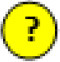	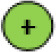	8/10
Demir and Sahin (33)	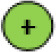	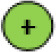	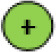	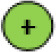	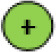	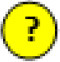	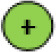	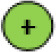	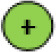	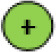	9/10
Fernández et al. (34)	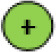	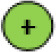	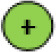	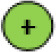	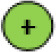	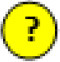	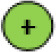	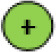	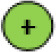	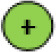	9/10
González-Gil et al. (35)	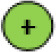	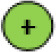	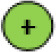	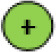	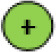	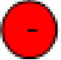	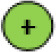	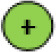	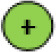	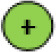	9/10
Gordon et al. (36)	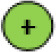	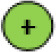	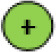	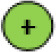	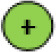	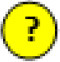	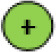	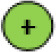	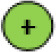	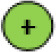	9/10
Green et al. (37)	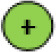	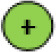	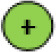	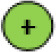	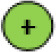	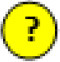	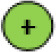	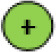	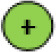	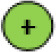	9/10
Hu et al. (38)	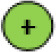	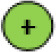	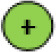	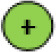	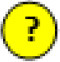	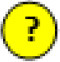	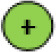	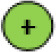	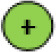	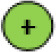	8/10
Koken et al. (39)	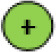	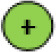	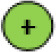	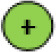	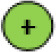	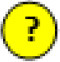	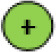	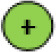	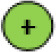	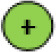	9/10
Lee et al. (40)	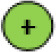	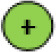	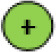	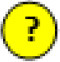	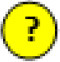	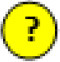	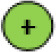	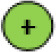	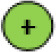	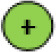	7/10
Moradi et al. (41)	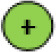	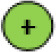	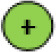	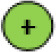	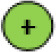	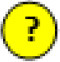	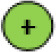	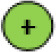	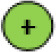	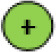	9/10
Moradi et al. (42)	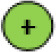	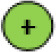	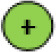	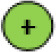	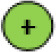	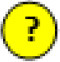	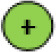	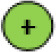	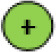	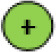	9/10
Rheaume et al. (43)	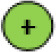	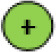	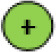	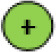	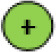	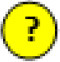	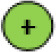	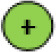	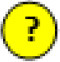	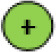	8/10
Sezgin et al. (44)	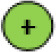	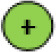	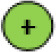	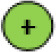	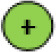	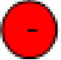	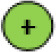	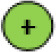	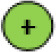	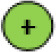	9/10
Ünver et al. (45)	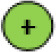	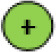	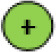	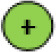	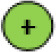	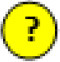	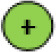	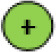	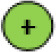	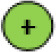	9/10
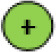 YES (clearly met)	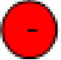 NO (clearly not met)						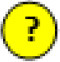 Can't Tell				
(A) Was there a clear statement of the aims of the research?											
(B) Is a qualitative methodology appropriate?											
(C) Was the research design appropriate to address the aims of the research?											
(D) Was the recruitment strategy appropriate to the aims of the research?											
(E) Were the data collection in a way that addressed the research issue?											
(F) Has the relationship between researcher and participants been adequately considered?											
(G) Have ethical issue been taken into consideration?											
(H) Was the data analysis sufficiently rigorous?											
(I) Is there a clear statement of findings?											
(J) How valuable is the research?											

### General characteristics of the selected studies

An overview of the included studies (*n* = 19) has been illustrated in Table 2. Nine articles were published in 2021 (29–32, 34, 36, 38, 41, 42) and 10 were published in 2022 (27, 28, 33, 35, 37, 39, 40, 43–45).

**Table 2:** Summary of characteristics of the studies selected for meta-synthesis.

**Authors, Country**	**Aim**	**Method**	**Data collection/ data analysis**	**Participant selection**	**Main finding**
Andersson et al. (27), Sweden	To investigate person-centered care based on ICU nurses' experiences during the first phase of the COVID-19 pandemic	Qualitative	Semi-structured interviews; Content analysis	Not specified; 6 ICU nurses	Prerequisites, care environment, person-centered processes, person-centered outcomes
Aydin et al. (28), Turkey	To describe the self-transcendence of the leading fighters, intensive care nurses, during the COVID-19 pandemic	Qualitative	Semi-structured interviews; Phenomenological approach	Snowball sampling; 25 ICU nurses	Improvement in nursing roles and skills, being proud of oneself and the team, understanding the value of life, physical and mental well-being, administrative loneliness, inability to give care, fear of being a source of infection, loneliness of patients, personal and contextual factors
Bergman et al. (29), Sweden	To describe Swedish registered nurses' experiences of caring for patients with COVID-19 in ICUs during the pandemic	Mixed method survey	Online questionnaire; Inductive content analysis	Convenience sampling; 282 ICU nurses	Tumbling into chaos, diminished nursing care, transition into pandemic ICU care
Cadge et al. (30), USA	To understand how nurses experience providing care for patients hospitalized with COVID-19 in ICUs	Qualitative	Semi-structured interviews; Thematic analysis	Purposive sampling; 16 ICU nurses	Challenges of maintaining existing working relationships, challenges of working with new co-workers and teams, Importance of institutional level acknowledgment of their work, role of nursing leadership in providing information and maintaining morale
Chegini et al. (31), Iran	To describe the experiences of ICU nurses caring for patients infected by COVID-19	Qualitative	Semi-structured interviews; Phenomenological approach	Purposive and snowball sampling; 15 ICU nurses	Organizational challenges, psychological challenges, professional challenges, social challenges
Conz et al. (32), Brazil	To understand the experiences of ICU nurses providing care to COVID-19 patients	Qualitative	Individual interviews; Social phenomenological approach	Snowball sampling; 20 ICU nurses	Adjusting to the new way of delivering care in ICU, being around situations that interfere with physical and mental health, projecting professional life after the COVID-19 pandemic
Demir and Sahin (33), Turkey	To explore the experiences of nurses providing care to ICU patients diagnosed with COVID-19	Qualitative	Semi-structured interviews; Descriptive phenomenological approach	Snowball sampling; 12 ICU nurses	Fear and anxiety compromise care, difficulties in caring for COVID-19 patients in ICU, coping with the difficulties in caring for COVID-19 patients in ICU
Fernández-Castillo et al. (34), Spain	To explore the experiences and perceptions of nurses working in an ICU during the COVID-19 global pandemic	Qualitative	Semi-structured interviews; Inductive content analysis	Purposive sampling; 17 ICU nurses	Providing nursing care, resources management and safety, psychosocial aspects and emotional lability, professional relationships and fellowship
González-Gil et al. (35), Spain	To describe the experience of nurses in caring for patients with COVID-19 in ICUs	Qualitative	Semi-structured interviews; Phenomenological approach	Purposive sampling; 17 ICU nurses	The value of human resources, it's not the beds, it's the expert staff, shouldering the patient's burden, suffering because they have not cared well
Gordon et al. (36), USA	To examine the experiences of ICU nurses caring for COVID-19 patients.	Qualitative	Semi-structured interviews; Content analysis	Purposive sampling; 11 ICU nurses	Emotions experienced, care environment challenges, physical symptoms, short term coping strategies, social effects
Green et al. (37), Israel	To explore the experiences of ICU nurses caring for COVID-19 patients who eventually died during	Qualitative	Semi-structured interviews; Descriptive phenomenological approach	Purposive sampling; 24 ICU nurses	The first vs. the second COVID-19 waves, fighting for life and being unable to win, a chronicle of pre-determined death, nurse's emotional coping with patient death
Hu et al. (38), China	To examine ICU nurses' experiences of caring for patients with COVID-19	Qualitative	Individual interviews; Descriptive phenomenological approach	Purposive sampling; 13 ICU nurses	Initial response, adaption, desperation, holding on and surviving
Koken et al. (39), Turkey	To understand the experiences of cardiovascular nurses working in a COVID-19 ICU during the pandemic	Qualitative	Semi-structured interviews; Phenomenological approach	Snowball sampling; 10 ICU nurses	The duties and responsibilities in a COVID-19 ICU, the differences of COVID-19 ICU practices from cardiovascular practices, the transferrable skills of cardiovascular nurses in a COVID-19 ICU, the difficulties encountered working in a COVID-19 ICU the difficulty of working with personal protective equipment, the psychosocial effects of working in a COVID-19 ICU
Lee et al. (40), Taiwan	To understand the perceived stress and coping behaviors of ICU nurses caring for critically ill patients with COVID-19	Mixed-method	Semi-structured interviews; Content analysis	Not specified; 85 ICU nurses	Fear and worry, increased burden, coping behavior
Moradi et al. (42), Iran	To explore the protective reactions of ICU nurses providing care for patients with COVID-19	Qualitative	Semi-structured interviews; Content analysis	Purposive sampling; 14 ICU nurses	Unbalanced self-protective reactions, responsible self-protective reactions
Moradi et al. (41), Iran	To explore the challenges experienced by ICU nurses throughout the provision of care for COVID-19 patients	Qualitative	Semi-structured interviews; Content analysis	Purposive sampling; 17 ICU nurses	Organization's inefficiency in supporting nurses, living with uncertainty, physical exhaustion psychological burden of the disease
Rhéaume et al. (43), Canada	To explore Canadian ICU nurse's experiences providing care to COVID-19 patients during the second wave of the pandemic	Qualitative	Online survey; Thematic analysis	Convenience sampling; 108 ICU nurses	Managing the pandemic, witness to families' grief, our safety, futility of care
Sezgin et al. (44), Turkey	To explore the experiences of the ICU nurses caring for patients diagnosed with COVID-19	Qualitative	Semi-structured interviews; Thematic analysis	Purposive and snowball sampling; 10 ICU nurses	Death and fear of death, impact on family and social lives, nursing care of COVID-19 patients, changing perceptions of their own profession: empowerment and dissatisfaction, experiences and perceptions of personal protective equipment and other control measures
Ünver et al. (45), Turkey	To understand the PPE-related skin changes experienced by ICU nurses working during the COVID-19 pandemic	Qualitative	Semi-structured interviews; Phenomenological approach	Convenience sampling; 14 ICU nurses	Main causes of PPE-related skin changes, the location of the skin changes caused by PPE, secondary adverse effects of PPE-related discomfort, symptomatology of PPE-related skin changes, prevention of PPE-related skin changes, therapeutic interventions for curing for PPE-related skin changes

Five studies were from Turkey (28, 33, 39, 44, 45) three from Iran (31, 41, 42), two from the USA (30, 36), two from Spain (34, 35), two from Sweden (27, 29), one from China (38), one from Israel (37), one from Brazil (32), one from Canada (43), and one from Taiwan (40). The included studies were conducted in various contexts, in terms of the healthcare system, the number of deaths and cases of COVID-19, the nurse-to-patient ratio, economic and political status, population age pyramid, culture, equipment, and access to health services. For example, the Iranian health system was faced with more hurdles than other countries, due to the sanctions imposed against the country. Furthermore, there are physician-dominant policies in the Iranian health system (31, 41, 42). The United States makes up < 5% of the world's population. Nevertheless, it leads the world in the number of cases of COVID-19 and deaths. In addition, there is considerable variation between different states (46). Spain has the oldest population and the highest life expectancy in the world (47). In Sweden, ICU nurses are registered nurses specializing in intensive care or anesthesia, and the nurse-patient ratio in the ICU is usually 1: 1–2, which was the case even before COVID-19 (29). Regarding the studies' methodologies, seventeen studies (27, 28, 30–39, 41–45) used a qualitative design and two had a mixed method design (29, 40).

### The experiences of ICU nurses working with COVID-19 patients

**Figure 2:**
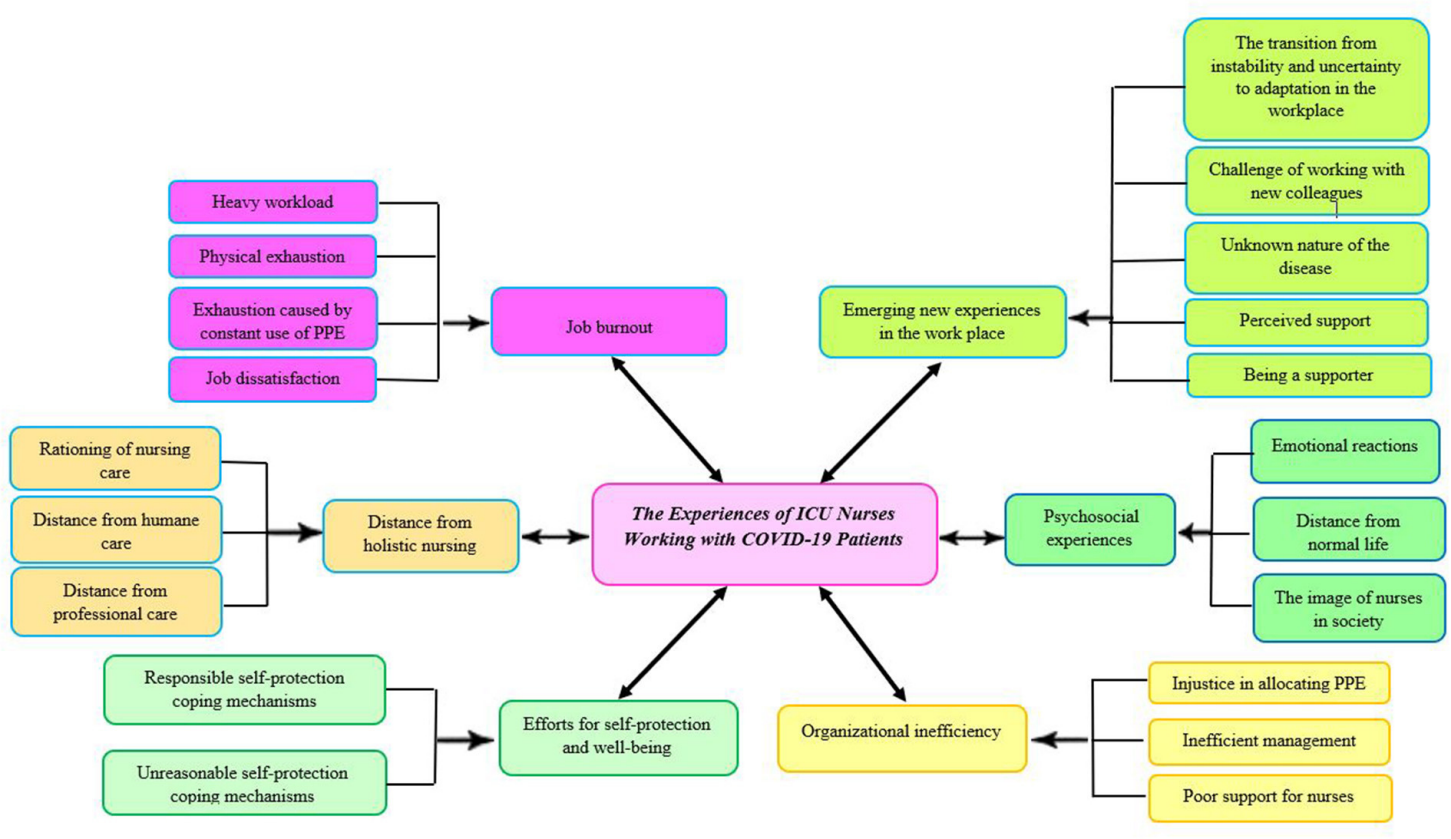
Experiences of intensive care unit nurses working with COVID-19 patients.

As the result of inductive content analysis, six main categories were identified regarding the experiences of ICU nurses working with COVID-19 patients: “distance from holistic nursing,” “psychosocial experiences,” “efforts for self-protection and wellbeing,” “organizational inefficiency,” “job burnout,” and “emerging new experiences in the workplace” (Figure 2).

#### Distance from holistic nursing

This review indicated that the number of patients admitted to the ICU increased during the COVID-19 pandemic. Nurses had less time to care for patients, and the nurses' interaction with infected patients was reduced due to the fear of being infected with COVID-19 and transmitting the virus to their loved ones, as well as due to the use of PPE. In addition, hospitals faced a shortage of skilled nurses specializing in intensive care. Therefore, most nurses felt that the care they provided during the COVID-19 pandemic had distanced from holistic care. Experiences related to distance from holistic nursing were divided into three subcategories, including rationing of nursing care, distance from humane care, and distance from professional care.

##### Rationing of nursing care

Rationing of nursing care refers to the failure to provide one or more of the required types of nursing services (48). Thus, nurses find it impossible to perform all nursing requirements, and in these situations, they may reduce, delay, or simply eliminate care (49). ICU nurses felt that they did not have enough time to perform all nursing care during the COVID-19 pandemic. They couldn't give patients the care that they normally gave them. Therefore, care, such as patient mobility, care of central and peripheral pathways devices, and primary care were not provided to patients, and a decrease in nursing care standards was reported. In addition, nurses prioritized maintaining airways, maintaining hemodynamic constants with medication, and administering essential medications to patients (27, 29, 34, 35, 42, 44).

“*We do not have the time to do all the things that we usually do, such as oral care or changing the patients posture in bed. Relatives, who normally are a resource, cannot even visit the patient until he or she is dying”* *(**29**)*.

##### Distance from humane care

Humane care preserves human dignity and value. Nurses must integrate humane care throughout patient care (50). Nevertheless, ICU nurses described the care provided to patients with COVID-19 as inhumane. These nurses experienced a lack of patient personalization, limited integration of patients in the ICU, ban on the presence of patient relatives, reduction or modification of their relationship with the patient, patients dying alone, and communication problems with patients (27–29, 34–36, 39, 42, 43).

“*It just feels more distant because you're gowned up you feel like you're in a suit all the time. You can't really make that personal connection, they can't see, you can't see, you're in a mask and glasses…”* *(**36**)*.

“*I should not be the last voice they (patients) hear. It should be someone they love”* *(**43**)*.

##### Distance from professional care

Nurses stated that they did not perform trained and specialized nursing duties and were restricted to the tasks of a physician's assistant. Therefore, they had feelings of inadequacy when providing care to patients (27–30, 34, 44).

“*The quality of nursing care (and the ICU care in general) is lower than usual.” “... it feels like we are just medical assistants who change the patient's infusions”* *(**29**)*.

“*…I just felt like sometimes I was just a body there, like I wasn't actually doing nursing things that I've been trained to do”* *(**30**)*.

#### Psychosocial experiences

Within patient care, ICU nurses endure psychological pressure. In the current pandemic, these pressures have intensified, as it has created tremendous demands and expectations, both physically and mentally, because COVID-19 is an emerging infectious disease with a high mortality rate and yet no absolute treatment. Furthermore, they often experienced stigma outside of the hospital due to providing direct care for COVID-19 patients (30, 36). Stigma is a social process identified by labeling, separation, and stereotyping. The experience of stigmatization can lead to negative psychosocial experiences and effects (51). Therefore, ICU nurses experienced various psychological issues. Psychosocial experiences were separated into three subcategories, including emotional reactions, distance from normal life, and the image of nurses in society.

##### Emotional reactions

The nurses reported contradictory feelings about work in ICUs. Almost all nurses experienced negative emotional reactions including stress, fear, and worry about being infected and infecting their loved ones (27–29, 31–34, 36, 38–40, 42). In addition, they felt anxiety, helplessness, pangs of conscience, feeling lonely (27, 28, 32, 36, 39), suffering, frustration (33–36, 38), and even aggression and angry (33, 42). Furthermore, they were very depressed and sad due to facing the patients' death (37).

“*But with these patients, because of the risk to myself (crying) and the risk of bringing home something to my family, it is very high stress”* *(**36**)*.

“*The stress caused by this disease has made me a little more aggressive, as I sometimes even become hostile toward my family, especially my brother”* *(**42**)*.

“*I mostly experienced fear… I think and fear that in the future myself or anyone in my family may encounter this disease”* *(**33**)*.

If we look at the COVID-19 pandemic from another perspective, we might see that it could create positive emotional reactions in nurses. Nurses tried to empathize with patients (28, 36). They enjoyed cooperating with colleagues and were happy with their patients' recovery (28, 29). In addition, with the decrease in the number of hospitalized patients, hope arose among nurses (38).

“*I learned to empathize with my patient, being in their shoes…That's what happened here, knowing his situation, knowing that he has kids that love him, care for him, the same level that I would love my parents”* *(**36**)*.

##### Distance from normal life

During the pandemic, nurses were subjected to discrimination, loneliness by others, and social isolation, in addition to intense work stress. Providing care to infected individuals, working in high-risk areas, and living in isolation from their family members, led to nurses feeling that their lives had become distanced from the normal route. Their families feared getting infected and obsessed over this (42). Therefore, nurses had to reduce interactions with their families and isolate from them (28, 29, 33, 34, 36, 39, 42, 44). They felt uncertainty in life (28, 34) negative behaviors, and a lack of understanding from friends and family members (30, 33).

“*Our lives have gone off-track, and we have no peace. Before the current pandemic, when we got home after the shift, we could at least cuddle our children. My wife and I would at least talk together, but not now! We cannot cuddle our children. Contacts are limited. We suspect and doubt anything and everything in our own home, which is the safest place in terms of Corona”* *(**42**)*.

“*...and I have a fear of infecting people around me, so I don't meet anyone”* *(**33**)*.

##### The image of nurses in society

During the COVID-19 pandemic, nurses have continued to provide care for infected patients, while, at the same time, many jobs were closed or were performed online. The efforts of nurses to save human lives and provide care have also been portrayed by the media and social media. Therefore, people have become more aware of the importance of nursing. The nurses' families have shown pride in them, and the public has shown appreciation for their efforts. Nurses have been portrayed as heroes and valuable health workers (28, 31, 35, 36, 38, 39). Thus, the COVID-19 pandemic has created a unique image of nurses in society.

“*I know that everybody's trying to be nice by calling us a hero but it's just like I don't feel that way, I never have felt that way. It doesn't feel like that, heroes are supposed to save everybody, we're not doing that”* *(**36**)*.

#### Efforts for self-protection and wellbeing

ICU nurses were more concerned than others because they were not only at the center of the crisis and at high risk of infection to themselves and their loved ones, but also faced problems such as lack of PPE and lack of organizational support. Consequently, they tried to save their lives and wellbeing by adopting various behaviors in order to stay safe and healthy. Their efforts toward self-protection and wellbeing contain two subcategories, including responsible self-protection coping mechanisms and unreasonable self-protection coping mechanisms.

##### Responsible self-protection coping mechanisms

Responsible self-protection was one of the nurses' coping mechanisms when providing care for patients with COVID-19. These mechanisms include willingness to perform laboratory tests (34), self-supplying PPE, purchasing high-quality PPEs on the open market in terms of skin and respiratory protection, and strict adherence to personal protection principles when providing care to patients with COVID-19. For example, they usually wore three pairs of gloves during performing procedures (38, 42, 45). Furthermore, they tried to maintain their physical and mental health and wellbeing through positive thinking and attitude, religious activities, interaction with family and friends, entertainment, adequate sleep and food, rest, seeking support, relaxation, shopping, and positive acceptance (28, 33, 40).

“*I wasn't afraid or scared of providing care for patients with COVID-19, but I was careful; fear leads to death. I was very cautious in providing care for these patients and carefully observed all principles of personal protection”* *(**42**)*.

##### Unreasonable self-protection coping mechanisms

Some nurses had unreasonable self-protection coping mechanisms. They displayed behaviors such as obsession with the infection, prolonged scrubbing, and doubt about the protocols (31, 42). Furthermore, some nurses considered only their own life and wanted to work in other wards. Some of them had challenges related to PPE and tried to steal PPE from other colleagues. In contrast, some others were indifferent to using PPE through self-censorship (42).

“*We've become far too obsessive about everything. When the shift is over, it takes an hour for us to leave the ward; we disrobe and scrub. Our scrub is frightening. We think everything is infectious because of our obsession. Interestingly, it is the same at home, too”* *(**42**)*.

#### Organizational inefficiency

Nurses felt unheard and unseen. They were dissatisfied with organizational inefficiency and poor support and expressed that the healthcare system was not ready for pandemic conditions. Organizations couldn't provide adequate support for nurses. Therefore, ICU nurses had to work in conditions such as a lack of PPE, improper PPE, and long shifts due to nursing shortage. Organizational inefficiency is divided into three subcategories including injustice in allocating PPE, inefficient management, and poor support for nurses.

##### Injustice in allocating PPE

Nurses complained about the doctor's dominance over the system and the discrimination of the authorities in allocating PPE (42).

“*Doctors are dominant here. Doctors are given the best gear, but it isn't like that for nurses. A nurse is condemned to work with any equipment they are given”* *(**42**)*.

##### Inefficient management

Inefficient management was another subtheme of organizational inefficiency. Nurses reported the nurse managers as unsupportive and invisible. They also described that managers' performance in training, supervising, providing manpower, and PPE for nurses was insufficient. The need to improve nursing skills and monitor nurses' performance was felt by nurses during the COVID-19 pandemic (27, 28, 30, 34). In addition, nurses faced problems such as a lack of preparedness of the health system to deal with the disease, nursing shortage (29, 31, 36, 40, 42, 43, 45), and lack and insufficient quantity and quality of PPE (27–29, 31, 34, 36, 40, 42, 43, 45).

“*There was no mask in the early days of the disease. We saw that disinfectant solutions were not in the ward and could not be found. The supply of gloves was reduced. Equipment was scarce”* *(**31**)*.

##### Poor support for nurses

Nurses expressed that they needed the support of the authorities. Nevertheless, they did not receive adequate support from officials. Ignorance by officials, cancellation of contracts in private hospitals, lack of financial support, respect, attention, and appreciation, and dissatisfaction with officials were also reported by nurses. In addition, they expected competence-based evaluation (27, 28, 30–32, 42–44).

“*Since the outbreak of Coronavirus, no university deputies or hospital managers have come to ask ‘What are you doing here? What kinds of problems are you facing?' this shows that the system is not much concerned about personnel”* *(**42**)*.

#### Job burnout

The highly contagious nature of COVID-19 increased patients' needs for intensive care. Nurses had to wear PPE for long periods of time. Furthermore, many nurses were infected and some of them unfortunately died. Accordingly, the workload of ICU nurses increased exponentially. Therefore, during the COVID-19 pandemic, ICU nurses were constantly faced with stressful conditions, which resulted in emotional exhaustion, while managing complex treatment and care processes. Working for long periods of time in an environment with a high level of uncertainty and stress, increasing workload, with nurses' units or wards being relocated, caused ICU nurses to experience burnout more rapidly. Furthermore, the inconsistency between the ideal expectations of the nursing profession and the situations faced in real life also increased burnout. As a result, these factors contributed to the high intention to leave jobs among ICU nurses. The job burnout category was segmented into four subcategories including heavy workload, physical exhaustion, exhaustion caused by the constant use of PPE, and job dissatisfaction.

##### Heavy workload

Working during the COVID-19 pandemic increased the nursing workload. Nurses had long shifts without a break and multiple responsibilities such as technical management of material, and managing human resources (27, 29, 31, 32, 34–36, 38, 40, 42, 43, 45).

“*There is a great responsibility for us with experience and competence in intensive care. We have to lead the work, support and teach our new colleagues, and at the same time be responsible for many patients besides those we care for ourselves”* *(**29**)*.

##### Physical exhaustion

Nurses experienced various signs and symptoms of physical exhaustion including, fatigue (29, 34, 36, 42), sweating (34, 36), vomiting and fainting (34), sleep disturbances (31, 36), headache and migraine (36), hard and heavy breathing (36), and spots and skin damage (42).

“*…The first time I took care of a patient with COVID-19 I couldn't stop sweating …”* *(**34**)*.

##### Exhaustion caused by constant use of PPE

Nurses have to wear PPE for long periods of time. Wearing PPE generates a lot of heat which is hard to bear. Nurses experienced many problems such as fatigue, difficulty breathing, hypotension, skin abrasions, sweating, headache, difficulty focusing, drinking, and eating due to wearing PPE. In addition, nurses spent a lot of time wearing PPE and it became more difficult for them to perform the procedure, thus their workload increased. Therefore, they described an uncomfortable feeling from wearing PPE (32, 33, 38–40, 42, 44, 45).

“*The clothes we wear make us very tired during the shift. Besides, with these on, we cannot eat or use the bathroom, especially during night shifts”* *(**42**)*.

“*Since we could not remove the equipment, we could neither drink water nor go to the toilet during the time we worked”* *(**33**)*.

##### Job dissatisfaction

Some nurses regretted being a nurse. Some quit or desire to quit their job and did not turn up for their shifts (32, 42, 44).

“*We were all unhappy about being a nurse, and wish we had another job that would take us away from this setting”* *(**42**)*.

As a result, heavy workload, physical exhaustion, exhaustion caused by the constant use of PPE, and job dissatisfaction contributed to high intention to leave jobs among ICU nurses.

#### Emerging new experiences in the workplace

The COVID-19 pandemic created new conditions in hospitals. In response to the current pandemic, new ICUs were established; nurses were transferred to new wards and new treatment teams were formed. Therefore, new experiences emerged in the ICU nurses' workplace. These experiences were separated into five subcategories, including the transition from instability and uncertainty to adaptation in the workplace, the challenge of working with new colleagues, the unknown nature of the disease, perceived support, and being a supporter.

##### The transition from instability and uncertainty to adaptation in the workplace

The COVID-19 pandemic created a standby situation for nurses. At the beginning of the pandemic, many changes were created in the structure of ICUs. Nurses were transferred to unfamiliar wards and were unsure of their duties. In addition, the condition of the patients was unstable and many of them died. Nurses stated it was like “being in a war zone” with patients dying despite their efforts to recover. Furthermore, the guidelines were constantly changing and the treatment process was different in different waves of the pandemic. Therefore, uncertainty and instability were the predominant experiences of ICU nurses. Nevertheless, with the overtime, the nurses stated that they became more patient than before, their management, planning, and practical nursing skills improved and they gained more experience in managing the pandemic, as a result, they gradually got used to the conditions. Therefore, adaptation and experience have replaced uncertainty (27–32, 36, 37, 40, 43, 44).

“*I think that there should have been more check ins with the nursing staff that got floated, for sure, because you took them from their comfort home, you took them from doctors they know, you took them from a layout of a floor that they know and you dumped them in a unit that you had no clue about”* *(**30**)*.

“*…I was on guard duty at the beginning of the pandemic and I felt so bad. I had a stomachache like a child starting elementary school. But now I am doing my best. I'm just not in this situation. Many people are in this situation. Frankly, I'm going a little more comfortably because I'm doing my best. I got a little more used to it. I also learned to integrate this into my life…”* *(**28**)*.

##### The challenge of working with new colleagues

In response to the increasing number of patients and ICU beds, new nurses were added to old care team and new teams were formed. As a result, nurses experienced a variety of challenges in working with new colleagues. They worked with new nurses on each shift and communication between new team members was poor. New nurses with no critical care experience started working in ICUs without receiving adequate training. In addition, nurses were uncertain about the qualifications of the new co-workers that they were also responsible for. Therefore, their workload increased (27, 29, 30, 34, 35, 39, 43, 44).

“*Many new colleagues with different experiences and competencies meant a greater responsibility for me [as an ICU-nurse]. Even if I ‘just' had to care for two or three patients, I also had to ensure that the other patients received appropriate care and support from my colleagues”* *(**29**)*.

“*The problem is that the ratio was a bit distorted because the nurse you were assigned as a companion could not take on the same as another nurse who knew the patient”* *(**35**)*.

##### The unknown nature of the disease

The unknown nature of the disease also affected nurses during the COVID-19 pandemic. The route of transmission, clinical signs, and prognosis of the disease was unknown and there were rumors about the disease (31, 33, 42).

“*You don't know its clinical picture either. Are fever, cough, and shortness of breath the actual signs or not? You don't really know. We have had many of such patients with none of these signs. One patient said that he only had diarrhea”* *(**42**)*.

“*...and it's not a disease we know of, after all; we're just learning things, so it's very backbreaking”* *(**33**)*.

##### Perceived support

Some nurses reported that they received emotional and practical support from their colleagues and the authorities, and, in some situations, they preferred colleagues' support over institutional resources. This support included teamwork, strengthening relationships between nurses, clinical support *via* email and follow-up email notes, daily tips for ICU nurses issued by the authorities, extending contracts in public hospitals, in-group meetings with head nurses to express concerns and problems (28, 30, 31, 34, 35, 38, 39), and providing PPE and training (44).

“*I think honestly the best thing that happened to me during COVID was I didn't realize like how great my like actual [home unit] coworkers were…everyone just came together so well and they were always there for me…we were always there for each other… I feel like everyone [was] such a family”* *(**30**)*.

“*However, when the rescue teams came and there were more medical staff, we felt that the pressure was not as great as before”* *(**38**)*.

##### Being a supporter

Nurses tried to support patients and their families by creating support teams fluent in the patients' language, countering rumors, accompanying the patient when dying, helping patients communicate with their families, and training and providing appropriate evidence (30–32, 34–36, 39, 43).

“*Early in the onset of the disease, there were many rumors, and one of our most important tasks when serving was to counter these rumors and provide appropriate evidence for patients”* *(**31**)*.

## Discussion

This systematic review and meta-synthesis integrated the findings of relevant studies and synthesized them to provide in-depth insight and knowledge regarding the experiences of ICU nurses working with COVID-19 patients. Nineteen studies included in the meta-synthesis were from ten countries. Six main categories were identified, including distance from holistic nursing, psychosocial experiences, efforts for self-protection and wellbeing, organizational inefficiency, job burnout, and emerging experiences in the workplace.

The results of this review demonstrated a distance from holistic nursing. Nursing is a holistic profession, and there is a strong commitment to the idea that all components of the individual must be considered when caring for a patient. Nurses need to provide nursing care by considering the patient as a complete individual and trying to meet the biological, psychological, social, relational, and spiritual needs of the patient (52). Nevertheless, during the COVID-19 pandemic, most nurses rationed nursing care and distanced themselves from humane and professional care due to the fear of infection and a heavy workload. Such conditions affect the safety of patients. One possible solution is to reorganize nursing care in heavy-workload conditions, such as an ongoing pandemic, so that qualified ICU nurses can provide patient care.

This study found that ICU nurses experienced varied psychosocial effects. Similarly, evidence from the SARS pandemic showed that a prevalence of post-traumatic stress disorder and depressive disorder among HCWs was common (53). Furthermore, the results of a cross-sectional study on 117 ICU nurses caring for COVID-19 patients showed that 74.4% of nurses had moderate-to-severe perceived stress and 17.7% of participants indicated a probable diagnosis of post-traumatic stress disorder (54). Moreover, a systematic review found nurses experienced fear, concern, and anxiety during a respiratory pandemic (5). Stress, fear, anxiety, and worry are negative psychosocial experiences related to concerns about family members, the risk of infection, and the unpredictability of the disease, during the COVID-19 pandemic (17, 55). During the COVID-19 pandemic, ICU nurses have been witnessing the death of patients, end of life, family distress, and physical and psychological suffering. Furthermore, they have had to handle complex therapeutic regimens and sophisticated technical equipment. Therefore, working in the ICU is a source of psychosocial problems for nurses (56). In this regard, given the key role of nurses in providing high-quality care for COVID-19 patients, attention to their mental health and wellbeing should be a priority for policymakers and health system planners. In addition, appropriate interventions should be performed to improve their psychological condition, particularly in serious pandemic situations (57). Different interventions could be introduced to improve mental health, for example, screening and assessing mental health status, access to mental health care services and early supportive interventions for high-risk nurses, designated rest periods, social support to reduce feelings of isolation, sufficient PPE for nurses to provide protection (58).

The results of this review have shown that nurses have made efforts toward self-protection. Similarly to our results, a qualitative study reported nurses had obsessive behaviors during the COVID-19 pandemic (3). Infectious diseases are a significant threat to HCWs and nurses experience the risk of infection and the possibility of transmission to others (59). ICU nurses are at high risk of infection with COVID-19, due to performing aerosol-generated procedures, such as cardiopulmonary resuscitation and suction (60). Striving for survival is an inherent trait in humans. Therefore, it seems that it is normal for ICU nurses to try to save their own lives, which could be jeopardized at any time.

Our findings indicated organizational inefficiency in supporting the nurses and in pandemic management. In addition, nurses reported uncertainty in the workplace, the challenge of working with new colleagues, the unknown nature of the disease, perceived support, and being a supporter. Consistent with our results, a meta-synthesis of previous pandemics found that cooperation and camaraderie among nurses increased. Their collaboration expanded by sharing experiences, supporting each other, and fostering a team spirit. Nevertheless, organizational unpreparedness, lack of PPE, shortage of nurses, and a heavy workload were the most important challenges in working during the pandemic (5). Nurses play an important role in health promotion for patients, especially during the COVID-19 pandemic. They can provide high-quality and safe care only if they have the appropriate quality of work-life and if they receive adequate support. Therefore, it is necessary that nurse managers, nurse leaders, and healthcare officials should increase efforts to ensure the quality of work-life, dignity, and nurses' support using appropriate economic policies (61).

Findings from our review suggest that most ICU nurses experienced job burnout following physical exhaustion, heavy workload, exhaustion caused by the constant use of PPE, and job dissatisfaction, during the COVID-19 pandemic. Job burnout is defined as the experience of fatigue over a long period of time and reduced levels of job motivation, due to excessive demands in the workplace (62). Similarly, nurses experienced burnout owing to the heavy workload during the MERS outbreak (63). In addition, the results of a meta-analysis study by Galanis et al. showed that the level of burnout in nurses was high during the COVID-19 pandemic (58). Furthermore, a systematic review suggested that HCWs during the COVID-19 pandemic experienced a heavy workload, due to increased working hours, staff shortages, increased paperwork, and the use of PPE (64). Therefore, the need to decrease ICU nurses' burnout is evident. In this regard, healthcare organizations can help nurses by decreasing working hours and paperwork, increasing staff, attracting volunteer and skilled nurses, and providing more leave.

### Limitations

To the best of our knowledge, this study is the first systematic review and meta-synthesis concerning the experiences of ICU nurses working with COVID-19 patients. However, our study had several limitations. Firstly, it only included studies published in English. Secondly, this systematic review and meta-synthesis only synthesized the experience of ICU nurses. Therefore, it is recommended that, in future studies, researchers review the experience of nurses and other HCWs working in other hospital wards. Thirdly, the development of COVID-19 vaccines may affect nurses' experience of caring for patients with COVID-19, and it is suggested that this be considered in future research.

## Conclusion

This systematic review and meta-synthesis focused on the experiences of ICU nurses working with COVID-19 patients. Given the critical role of ICU nurses in the care process of COVID-19 patients, they require adequate attention and support. The findings from this review suggest healthcare authorities and policymakers can facilitate the provision of high-quality patient care during the COVID-19 pandemic through appropriate planning to provide adequate support and training, prevent shortages of nursing staff and equipment, and provide adequate attention to the psychological needs and job satisfaction of ICU nurses.

## Data availability statement

The original contributions presented in the study are included in the article/supplementary material, further inquiries can be directed to the corresponding author.

## Author contributions

All authors listed have made a substantial, direct, and intellectual contribution to the work and approved it for publication.

## Conflict of interest

The authors declare that the research was conducted in the absence of any commercial or financial relationships that could be construed as a potential conflict of interest.

## Publisher's note

All claims expressed in this article are solely those of the authors and do not necessarily represent those of their affiliated organizations, or those of the publisher, the editors and the reviewers. Any product that may be evaluated in this article, or claim that may be made by its manufacturer, is not guaranteed or endorsed by the publisher.
